# Altered Cerebral Blood Flow Covariance Network in Schizophrenia

**DOI:** 10.3389/fnins.2016.00308

**Published:** 2016-06-30

**Authors:** Feng Liu, Chuanjun Zhuo, Chunshui Yu

**Affiliations:** ^1^Department of Radiology and Tianjin Key Laboratory of Functional Imaging, Tianjin Medical University General HospitalTianjin, China; ^2^Department of Psychiatry Functional Neuroimaging Laboratory, Tianjin Mental Health Center, Tianjin Anding HospitalTianjin, China; ^3^Department of Psychiatry, Tianjin Anning HospitalTianjin, China

**Keywords:** schizophrenia, arterial spin labeling, cerebral blood flow, covariance network, small world, efficiency

## Abstract

Many studies have shown abnormal cerebral blood flow (CBF) in schizophrenia; however, it remains unclear how topological properties of CBF network are altered in this disorder. Here, arterial spin labeling (ASL) MRI was employed to measure resting-state CBF in 96 schizophrenia patients and 91 healthy controls. CBF covariance network of each group was constructed by calculating across-subject CBF covariance between 90 brain regions. Graph theory was used to compare intergroup differences in global and nodal topological measures of the network. Both schizophrenia patients and healthy controls had small-world topology in CBF covariance networks, implying an optimal balance between functional segregation and integration. Compared with healthy controls, schizophrenia patients showed reduced small-worldness, normalized clustering coefficient and local efficiency of the network, suggesting a shift toward randomized network topology in schizophrenia. Furthermore, schizophrenia patients exhibited altered nodal centrality in the perceptual-, affective-, language-, and spatial-related regions, indicating functional disturbance of these systems in schizophrenia. This study demonstrated for the first time that schizophrenia patients have disrupted topological properties in CBF covariance network, which provides a new perspective (efficiency of blood flow distribution between brain regions) for understanding neural mechanisms of schizophrenia.

## Introduction

Schizophrenia is a prevalent and costly psychiatric disorder that affects 1% of the population (Lewis and Lieberman, 2000). Because most of energy supplied to the brain is consumed to support spontaneous activity (Raichle and Mintun, 2006), many researchers have used resting-state functional MRI (fMRI) to investigate spontaneous activity in schizophrenia. They found that schizophrenia patients show intra-regional activity abnormality (Huang et al., 2010), inter-regional connectivity alteration (Wang et al., 2014) and functional network disruption (Guo et al., 2014). However, their pathophysiological interpretations face challenges because blood oxygenation level dependent (BOLD) signal is an indirect measure of neuronal activity and its changes depend on multiple factors, including metabolic rate of oxygen, cerebral blood volume, and cerebral blood flow (CBF) (Buxton et al., 2004).

In contrast to BOLD-fMRI, arterial spin labeling (ASL) measures CBF, a metric with definite physiological significance (Ye et al., 2000; Vaishnavi et al., 2010). ASL studies have found local CBF changes in several brain regions in schizophrenia, including the prefrontal, occipital, hippocampal regions, and basal ganglia (Pinkham et al., 2011; Walther et al., 2011; Liu et al., 2012; Zhu et al., 2015). In addition, schizophrenia patients have shown CBF connectivity changes, including increased connectivity in the precuneus of the default mode network (Kindler et al., 2015) and decreased connectivity between the left insula and postcentral gyrus and between the left thalamus and right medial superior frontal gyrus (Zhu et al., 2015). However, little is known about topological alterations of CBF network in schizophrenia.

Graph theory is a powerful method for delineating network topology of the brain, where the brain is depicted as a graph consisting of nodes (brain regions) and edges (connections) (Bullmore and Sporns, 2009; Wang et al., 2010). The small-world network is defined to have high degree of clustering and similar characteristic path length relative to the random network (Watts and Strogatz, 1998). This network supports both segregated and distributed information processing (Bassett and Bullmore, 2006), which is analogous to functional segregation and integration of the brain. In fact, small-world organization has been repeatedly reported in functional (Salvador et al., 2005) and anatomical networks (Hagmann et al., 2008) of human brain, and topological organizations of these networks are disrupted in schizophrenia (Liu et al., 2008; Wang et al., 2012).

In addition to functional and anatomical networks that constructed at the individual level, there is a growing interest in investigating the brain covariance networks constructed across subjects (i.e., group level), such as structure covariance network (SCN) based on gray matter volume (Bassett et al., 2008) or cortical thickness (He et al., 2007). The SCN has emerged as a powerful tool to study the human brain, providing complementary information to other connectivity approaches. Moreover, Gong et al. suggest that cortical thickness correlations partly reflect underlying anatomical connectivity and include unique information that represents a vital aspect of interregional relationship (Gong et al., 2012). In recent years, functional covariance network (FCN) method has attracted increasing attention. For instance, Di et al. (2012) report that the metabolic covariance patterns as revealed by fluorodeoxyglucose positron emission tomography (FDG-PET) data could partially reflect functional connectivity as revealed by resting-state fMRI. This study also suggests that investigation of the CBF covariance could bridge the gap between brain structure covariance to neural covariance. Melie-Garcia et al. (2013) subsequently construct the single-photon emission computed tomography (SPECT) derived CBF covariance network and examine the topological properties of the network in healthy subjects. However, the SPECT technique is limited by the use of invasive radioactive tracers and relatively low spatial resolution of the image.

Here, we used a noninvasive ASL technique and graph theoretical approach to investigate the topological organization of the CBF covariance networks in patients with schizophrenia and demographically matched healthy controls. We aimed to investigate whether and how topological properties of CBF covariance network are changed in schizophrenia, which may improve our understanding on neural mechanisms of schizophrenia from the perspective of efficiency of blood flow distribution between brain regions.

## Materials and methods

### Subjects

The present study was approved by the Ethics Committee of the Tianjin Medical University General Hospital. Written informed consent was obtained from each participant before the experiments. Ninety-six schizophrenia patients were recruited, and diagnoses of schizophrenia were determined by trained psychiatrists using the Structured Clinical Interview for DSM-IV (SCID, patient edition). The Positive and Negative Symptom Scale (PANSS) was used to evaluate severity of symptoms (Kay et al., 1987). Among them, 89 patients were treated with atypical antipsychotic medications when MRI examinations were performed. Exclusion criteria included participants with ages younger than 16 or older than 60 years, left-handedness, poor imaging quality, MRI contraindication, histories of CNS disorders, systemic illnesses, or substance abuse. Ninety-one age- and gender-matched healthy controls were recruited from the local community by advertisements and screened using the SCID (Non-patient edition) to confirm the current absence of any mental disorders. In addition, we excluded healthy subjects whose first degree relatives had any mental disorders.

### MRI data acquisition

MRI data were acquired using a 3.0-Tesla scanner (Discovery MR750, General Electric, Milwaukee, WI, USA). Tight but comfortable foam padding and earplugs were used to minimize head movement and to reduce scanner noise, respectively. The resting-state perfusion imaging was acquired using a pseudo-continuous ASL sequence with a three-dimensional fast spin-echo acquisition and background suppression. The tagging plane is 24 mm below the imaging slab, which is chosen to meet two criteria: (1) the gap between tagging plane and imaging slab as short as possible to guarantee the homogeneity of B1/B0 field, and (2) no magnetization transfer affection. Scan parameters were: repetition time/echo time = 4886/10.5 ms, Post-label delay = 2025 ms, spiral in readout of eight arms with 512 sample points, field of view = 240 × 240 mm^2^, flip angle = 111°, reconstruction matrix = 128 × 128, number of excitation = 3, in-plane resolution = 1.9 × 1.9 mm^2^, slice thickness = 4 mm, no gap and 40 axial slices. The total acquisition time for the ASL scan was 4 min and 44 s. All images were visually inspected to ensure them being free of visible image artifacts.

### CBF calculation and data preprocessing

The CBF values were quantified using the following equation:

$$\begin{array}{l} CBF\;=\;\frac{\rho_{b}\left(S_{c}-S_{l}\right)}{2\alpha C\omega_{a}\text{T}1_{\text{a}}\text{exp}\left(-\frac{\text{w}}{\text{T}1_{\text{a}}}\right)\left(1-\text{exp}\left(-\frac{\text{tl}}{\text{T}1_{\text{a}}}\right)\right)} \end{array}$$

where ρ_*b*_ represents the density of brain tissue, α represents labeling efficiency, *C* represents the sensitivity of the image to water, *w* represents the Post-labeling delay, *tl* represents the labeling duration, T1_**a**_ represents the T1 of arterial blood, ω_*a*_ represents the density of water in blood, and *S*_*c*_ and *S*_*l*_ represent signal intensities in the control and labeled images, respectively. For details of CBF calculation, see a previous study (Xu et al., 2010). The CBF maps were normalized to standard Montreal Neurological Institute (MNI) space by using the following three steps: (1) the native ASL images of the healthy controls were nonlinearly normalized to a standard perfusion template provided by SPM8 software (http://www.fil.ion.ucl.ac.uk/spm/software/spm8/) and then averaged to generate a study-specific standard ASL template; (2) all the native ASL images were nonlinearly normalized to this study-specific ASL template, and (3) all the CBF images were normalized to the standard space using the normalization parameters estimated from step (2) and resampled to a voxel size of 2 × 2 × 2 mm^3^. For standardization purpose, the CBF value of each voxel was divided by the mean CBF value of the whole brain (Liu et al., 2013).

### Weighted CBF covariance network

A network is comprised of nodes and edges. Here, nodes represent brain regions and edges represent statistical interdependence of CBF between nodes. The automated anatomical labeling (AAL) template (including 90 cerebral and 26 cerebellar regions) was used to divide the brain, and each region was defined as a node (Tzourio-Mazoyer et al., 2002). In line with previous small-world network studies in schizophrenia (Liu et al., 2008; Wang et al., 2012), we just focused on cerebral regions in the current study, and thus there were totally 90 nodes. The mean CBF value in each node was extracted for each subject, and a linear regression was performed at each node to remove the effects of age and gender. Pearson's correlation coefficients between the residuals of each pair of nodes across all subjects were considered as edges (Liu et al., 2015b). This procedure resulted in a 90 × 90 correlation matrix for each group.

This weighted network can utilize strength information of CBF coupling to better characterize network topology than the binary network. A sparsity threshold defined as the fraction of the total number of existing edges divided by the maximum possible number of edges, was applied to correlation matrices to minimize influence of intergroup difference in overall correlation strength and to enable all networks to have the same number of edges (Zhang J. et al., 2011). Because sparsity threshold selection may affect results of network analysis, we calculated network properties over a wide range of sparsity thresholds, which were selected using the following criteria: the average degree (the degree of a node is the number of connections linked to the node) over all nodes of each network was larger than log(*N*) (Watts and Strogatz, 1998; Achard et al., 2006), where *N* is the number of nodes (here, *N* = 90); and the resultant network had sparse and distinguishable properties compared to the degree-matched random network (He et al., 2007; Wang L. et al., 2009). Based on these criteria, the sparsity levels were ranged from 0.05 to 0.50, with a step of 0.01.

### Small world properties

At each sparsity threshold, we calculated global and nodal network properties. The global measures included (1) small-world parameters involving normalized clustering coefficient γ (a ratio of the clustering coefficient between real and 100 random networks, which quantifies the local interconnectivity of a network), normalized characteristic path length λ (a ratio of the characteristic path length between real and 100 random networks, which quantifies the overall routing efficiency of a network), and small-worldness σ = γ∕λ (measures the small-worldness of a network); and (2) network efficiency involving global efficiency E_glob_ (measures the ability of parallel information transmission over the network) and local efficiency E_loc_ (measures of the fault tolerance of the network). Of note, the characteristic path length was measured by harmonic mean distance between all possible pairs of nodes to overcome the problem from possibly disconnected network components. We also computed nodal properties, including the degree, efficiency, and betweenness of each node. Interpretation of these network measures please see Rubinov and Sporns (2010). The area under the curve (AUC) at different sparsity thresholds was calculated to provide an integrated scalar for each network measure.

### Statistical analysis

Permutation test was used to compare intergroup differences in AUC of each global network measure. For a given measure, we first calculated real intergroup difference of the measure. All subjects were then randomly reassigned to each group keeping the number of subjects in each group unchanged, and repeated network construction and small-world property calculation. The permutation process was repeated 1000 times and we counted the number of times that network measure difference in permutations was higher than real difference (Liu et al., 2015a). After dividing by the total number of permutations, the *p*-value was obtained for each measure. The significant level was set at *p* < 0.05 for each test.

Using this permutation framework, we also compared nodal properties between schizophrenia patients and healthy controls. Multiple comparisons were corrected using a false-positive correction *p* < 1/*N*, where *N* = 90 corresponds to the number of comparisons. This implied that we expected less than one false-positive per analysis (Lynall et al., 2010).

### Validation analysis

To test the effect of network types on our results, we performed the same analyses on the binary network as on the weighted network. Because the number of nodes may affect network analysis (Wang J. et al., 2009; Zalesky et al., 2010), we also used a high-resolution template with 1024 nodes (Zalesky et al., 2010; Zhang et al., 2011a) to construct CBF weighted covariance network. Given that there is a highly computational burden and random parcellation nature of the atlas, we only analyzed global small-world parameters.

## Results

### Demographic and clinical data of subjects

A total of 96 schizophrenia patients and 91 healthy controls were finally included. The two groups were matched for gender (42 males and 54 females for the patient group; 43 males and 48 females for the control group; *p* = 0.63) and age (33.57 ± 8.64 years for the patient group; 33.35 ± 10.44 years for the control group; *p* = 0.87). The positive, negative and general PANSS scores for schizophrenia patients were 16.78 ± 7.86, 20.04 ± 8.86 and 34.01 ± 10.78, respectively. The detailed demographic and clinical data are presented in Table 1.

**Table 1:** **Demographic and clinical characteristics of subjects**.

**Variables (Mean ± SD)**	**Schizophrenia**	**HC**	***P*-values**
Gender (M/F)	42/54	43/48	0.63^a^
Age (years)	33.57 ± 8.64	33.35 ± 10.44	0.87^b^
Duration (months)	121.38 ± 97.30	–	–
**PANSS**			
Positive score	16.78 ± 7.86	–	–
Negative score	20.04 ± 8.86	–	–
General score	34.01 ± 10.78	–	–

a *The p-value was obtained by chi-square test*.

b *The p-value was obtained by two sample t-test*.

*F, female; HC, healthy controls; M, male; PANSS, positive and negative symptom scale; SD, standard deviation*.

### Global topological measures of the network

**Figure 1:**
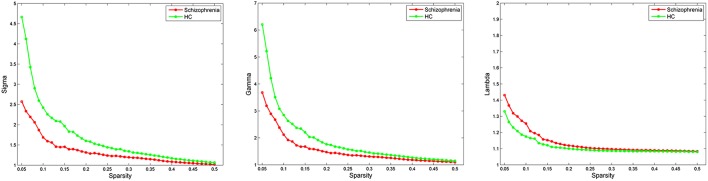
**Small-world parameters of CBF covariance network as function of sparsity thresholds**. Both the schizophrenia and control groups show a small-worldness (σ) >1, normalized clustering coefficient (γ) >1 and normalized characteristic path length (λ) approximately equal to 1, indicating that both groups exhibited a small-world topology. HC, healthy controls.

**Figure 2:**
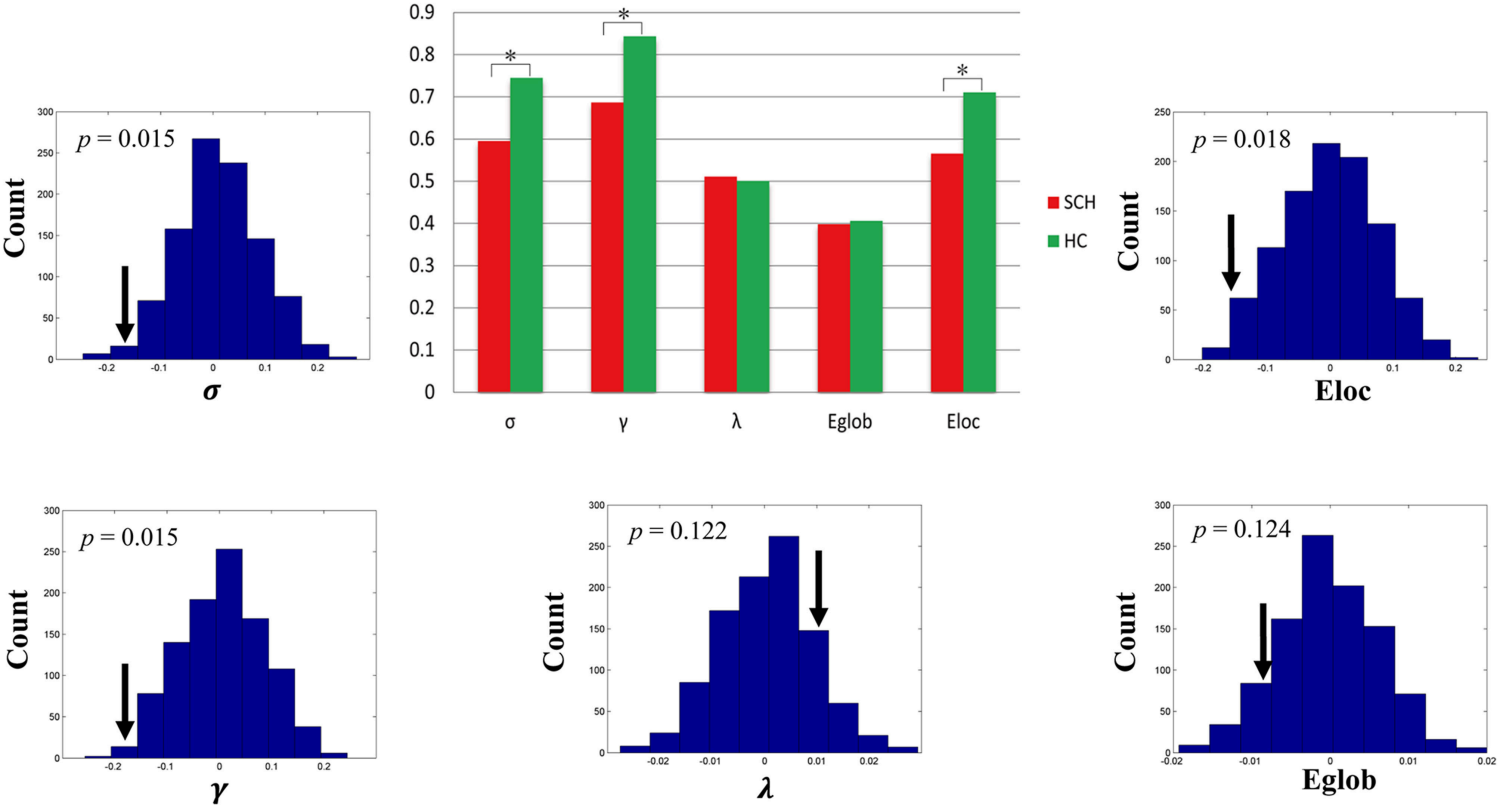
**Global topological differences in CBF covariance network between schizophrenia patients and healthy controls**. Each measure is expressed as the integrated area under the curve (AUC). The black stars in bar plots denote statistically significant differences between the two groups (permutation test, *p* < 0.05). The histogram plots around the bar plots are null distributions of permutation tests of global network measures and the real measures are marked with black arrows. SCH, schizophrenia; HC, healthy controls.

Both schizophrenia patients and healthy controls showed typical small-world topology, i.e., the CBF covariance networks had larger clustering coefficients (γ) and almost identical shortest path lengths (λ) compared with matched random networks (Figure 1). However, schizophrenia patients had decreased small-worldness, normalized clustering coefficient and local efficiency, and unchanged normalized characteristic path length and global efficiency relative to healthy controls (Figure 2).

### Nodal topological measures of the network

**Figure 3:**
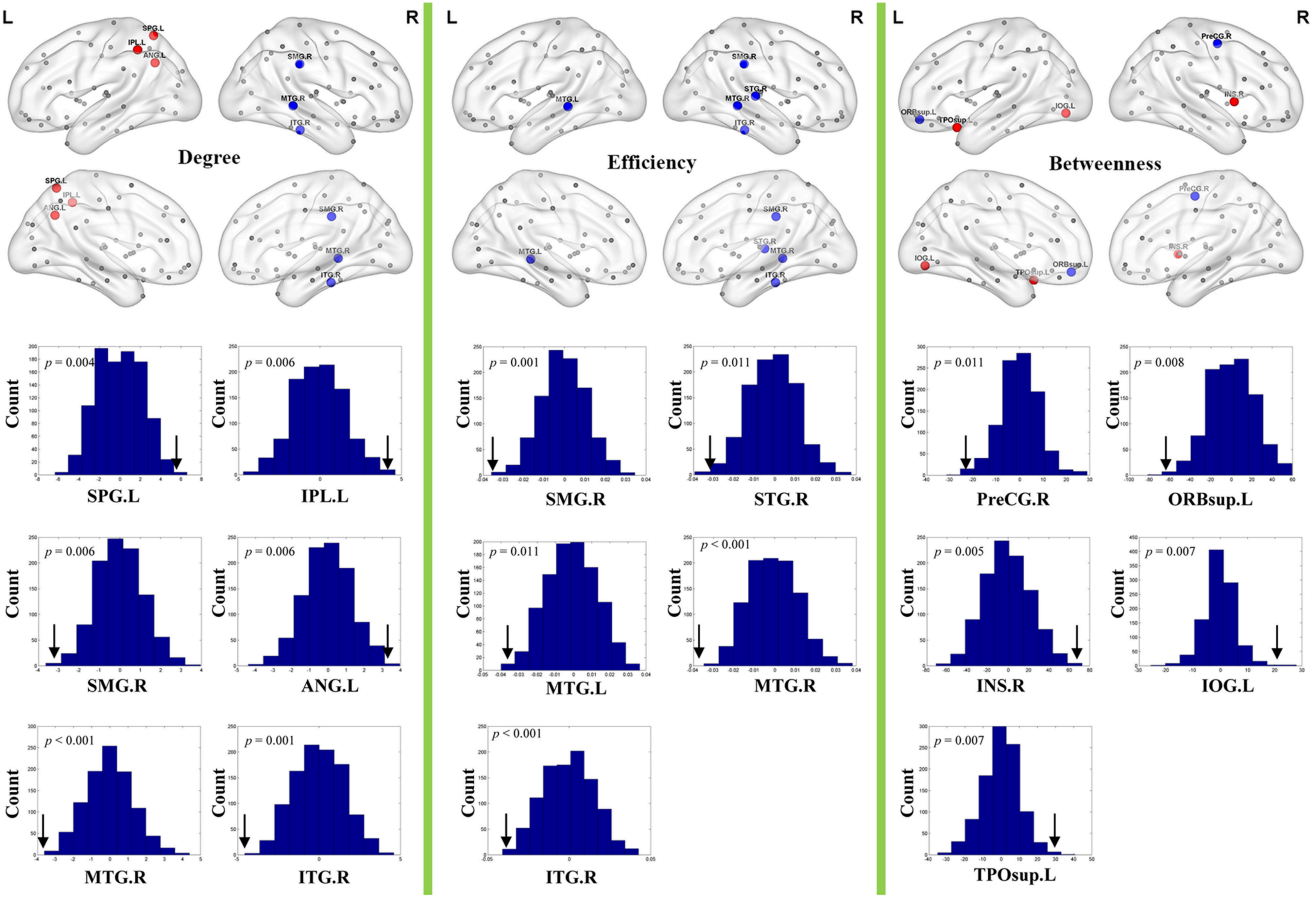
**Nodal topological differences in CBF covariance networks between schizophrenia patients and healthy controls (permutation test, ***p*** < 1/90)**. Results are rendered using the BrainNet viewer (Xia et al., 2013). Red and blue spheres represent regions with significantly increased and decreased nodal properties in schizophrenia, respectively. Gray spheres represent regions without significant intergroup difference. The histogram plots below the render plots are the null distributions of permutation tests of significant nodal network measures and the real measures are marked with black arrows. ANG, angular gyrus; INS, insula; IPL, inferior parietal lobule; IOG, inferior occipital gyrus; ITG, inferior temporal gyrus; L, left; MTG, middle temporal gyrus; PreCG, precentral gyrus; R, right; ORBsup, orbital part of superior frontal gyrus; SMG, supramarginal gyrus; SPL, superior parietal lobule; STG, superior temporal gyrus; TPOsup, superior part of temporal pole.

Brain regions exhibiting intergroup differences at least in one nodal property are shown in Table 2 and Figure 3. Compared with healthy controls, schizophrenia patients showed decreased nodal centrality (degree, efficiency, or betweenness) in the left orbital part of the superior frontal gyrus, the right precentral, supramarginal, superior and inferior temporal gyri, and the bilateral middle temporal gyri; and increased nodal centrality in the right insula, and the left superior and inferior parietal lobules, inferior occipital gyrus, angular gyrus, and superior part of the temporal pole.

**Table 2:** **Brain regions with altered nodal properties of CBF covariance network in schizophrenia**.

	***P* values**		
**Regions**	**Degree**	**Efficiency**	**Betweenness**
**Schizophrenia** > **HC**			
Right insula	–	–	0.005
Left inferior occipital gyrus	–	–	0.007
Left superior parietal lobule	0.004	–	–
Left inferior parietal lobule	0.006	–	–
Left angular gyrus	0.006	–	–
Left temporal pole, superior part	–	–	0.007
**Schizophrenia** < **HC**			
Right precentral gyrus	–	–	0.011
Left superior frontal gyrus, orbital part	–	–	0.008
Right supramarginal gyrus	0.006	0.001	–
Right superior temporal gyrus	–	0.002	–
Left middle temporal gyrus	–	0.011	–
Right middle temporal gyrus	<0.001	<0.001	–
Right inferior temporal gyrus	0.001	0.001	–

*Regions were considered abnormal in the patients with schizophrenia if they exhibited significant between-group differences in at least one of the three nodal topological characteristics. HC, healthy controls*.

### Validation analyses

We found that our main results were preserved in validation analyses of the binary network (Figures S1–S3, Table S1) and the weighted network constructed by 1024 nodes (Figures S4, S5).

## Discussion

This study used graph theory to investigate topological changes of CBF covariance network in schizophrenia. Both groups exhibited a small-world topology, implying an optimal balance between global and local information processing. At the global level, schizophrenia patients showed reduced small-worldness, normalized clustering coefficient and local efficiency, suggesting a shift toward random topology in CBF covariance network. At the nodal level, schizophrenia patients had altered nodal properties in the frontal, parietal, temporal, and occipital regions. These findings suggest imbalance in blood flow distribution between brain regions in schizophrenia, which indicates low efficiency of energy supply in schizophrenia.

Small-world topology is characterized by dense local connections with few long-range connections mediating short path length between distant pair of nodes in the network. This topology has both high global and local efficiency at low wiring cost (Bullmore and Sporns, 2009), making the brain to obtain an optimal balance between segregated and integrated information processing (Bassett and Bullmore, 2006). Small-world topology is an important organization of human brain and presents in various brain networks, including structural, anatomical, functional, metabolic, and electrical networks (Salvador et al., 2005; Iturria-Medina et al., 2008; Liu et al., 2008; Jhung et al., 2013; Hu et al., 2015). In line with these findings, we found that both schizophrenia patients and healthy controls showed small-world topology in CBF covariance network, indicating that the human brain irrespective of disease states is organized into small-world topology to support efficient information processing.

Although schizophrenia patients showed small-world topology in CBF covariance network, organizational efficiency of the network would be compromised in schizophrenia. In this study, schizophrenia patients had a decreased small-worldness, suggesting inefficient topological organization of CBF covariance network in schizophrenia. After scrutinizing topological properties, we found patients showing reduced local efficiency and unchanged global efficiency. Local efficiency is predominantly related to short-range connections between nearby regions that regulate the modularized information processing or fault-tolerance of a network (Latora and Marchiori, 2001); while the global efficiency is associated with information transfer between the remote cortical regions, and it is mainly linked with long-range connections. Thus, altered local but maintained global efficiencies may reflect that the impaired CBF covariance mainly concentrates on short-range rather than long-range covariance, which is also observed in functional connectivity studies (Chen et al., 2015). Based on these two measures, one can assess deviation of the patients' network from small-world topology, and particularly, suggest a shift toward random network architecture in schizophrenia. Because blood is the main source of energy supply to the brain, the inefficient CBF covariance network suggests low efficient energy supply in schizophrenia.

In addition to global properties, we also assessed nodal properties of CBF covariance network. Nodal degree, efficiency, and betweenness reflect the importance of a node in the network from different perspectives. We found altered nodal properties in regions involving in perceptual (i.e., precentral gyrus, inferior occipital gyrus, superior temporal gyrus, middle temporal gyrus, and inferior temporal gyrus), affective (i.e., insula, temporal pole, and orbitofrontal cortex), lingual and spatial processing (i.e., angular gyrus, supramarginal gyrus, and superior parietal lobule). These functional impairments have been frequently reported in schizophrenia (Bellani et al., 2009; De Sanctis et al., 2013; Postmes et al., 2014; Agarwal et al., 2015; Strauss et al., 2015). These findings are consistent with the concept that schizophrenia is linked with disturbance of multiple systems (Karbasforoushan and Woodward, 2012).

Although weighted edges can more accurately delineate the brain network than binary ones, it may be sensitive to noise. We found similar differences in binary network topological measures between the two groups, suggesting independency of our findings on network types. Furthermore, previous studies have shown dependence of topological measures of brain functional and anatomical networks on node numbers (Wang J. et al., 2009; Zalesky et al., 2010). To test the effect of node numbers on CBF covariance network topology, we repeated network analyses with 1024 nodes (Zalesky et al., 2010). We found that the overall patterns of network topological differences between the two groups were similar, suggesting the robustness of our results.

As a matter of fact, functional and anatomical connectivity networks are the two types of most widely used networks. In the past few years, there is growing evidence that SCN is a valuable avenue to explore the human brain (He et al., 2007; Zielinski et al., 2010; Alexander-Bloch et al., 2013; Evans, 2013). Recently, several researches suggest that investigation of FCN could also provide complementary yet crucial information for deep understanding the human brain. For example, Zhang et al. (2011b) examine inter-subject covariance of regional spontaneous activity and observe similar networks of the task-positive and task-negative networks as resting-state fMRI studies. Di and colleagues report that the covariance patterns of the brain metabolism partially reflect resting-state functional connectivity (Di et al., 2012). In consideration of the SCN partly mirroring underlying anatomical connectivity, we speculated the FCN has a close relationship with functional connectivity. On the other hand, BOLD fMRI is an indirect measure of neuronal activity and provides a relative measure of blood perfusion by measuring differences in oxygen consumption (Kim and Ogawa, 2012). Instead, CBF is a quantitative and absolute measure that closely links with neuronal activity. Thus, systematically analyzing the CBF covariance network may consolidate the foundation of findings observed in resting-state BOLD fMRI. Up to now, several researches have demonstrated altered small-world networks in schizophrenia by using resting-state fMRI. For instance, Liu et al. (2008) demonstrate disrupted small-world networks in schizophrenia, including significantly decreased small-worldness, normalized clustering coefficient, and local efficiency. Similarly, Lynall et al. (2010) observe reduced clustering and small-worldness in functional connectivity networks. Our results in the present study were consistent with previous findings, which provided further evidence of topological alterations of functional brain networks in schizophrenia.

It is worthy to note that a prior study has investigated the ASL-derived CBF networks using graph theoretical approaches (Liang et al., 2014). However, the method to construct CBF networks in their study is different from the current study, which is largely due to the different ASL sequence. Liang and colleagues used a 3D GRASE pCASL sequence and 120 images (60 label/control pairs) were acquired. Thus, they could obtain 60 CBF images for each subject and construct the CBF network at the individual level (across different CBF images of this subject). By contrast, we adopted a pseudo-continuous ASL sequence with a three-dimensional fast spin-echo acquisition. The signal to noise ratio of each image would be greatly improved but the acquisition time of each image is long. There were just 6 images (3 label/control pairs) and thus 3 CBF images were obtained. The final CBF image for each participant was generated by averaging the 3 CBF images and the CBF network was constructed at the group level (across subjects).

There were several limitations of the current study that merit consideration. First, most of the patients were chronic schizophrenia with mixed symptoms and had been received prescribed antipsychotic medications. Accordingly, we cannot rule out the influence of these factors on CBF covariance network. In the future, a large cohort of first-episode, drug-naïve patients with schizophrenia should be analyzed to validate our findings. Second, all covariance networks can only be constructed at the group level (He et al., 2007; Zielinski et al., 2010), we cannot examine relationships between topological properties and clinical parameters. Third, CBF covariance may reflect synchronization of blood flow distribution between brain regions; however, the biological meaning of CBF covariance needs to be further explored. Fourth, intravascular artifacts would inevitably affect CBF values. Although inflow saturation is implemented to suppress the intravascular signal results from the inflow blood during post labeling delay, we cannot fully exclude the influence of this issue to the network properties. Finally, the phase tracking errors is inherent to pseudo-continuous ASL (Jung et al., 2010; Fazlollahi et al., 2015), which will cause systematic biases. This issue was listed as a limitation of this study.

## Author contributions

FL, CZ, and CY designed the study. CZ acquired and FL analyzed the data. FL, CZ, and CY wrote the article, which all authors reviewed and approved for publication.

### Conflict of interest statement

The authors declare that the research was conducted in the absence of any commercial or financial relationships that could be construed as a potential conflict of interest.
