# Supplementary material for: Altered Cerebral Blood Flow Covariance Network in Schizophrenia
Source: Front Neurosci. 2016 Jun 30;10:308. doi: 10.3389/fnins.2016.00308 (PMC4927570; doi:10.3389/fnins.2016.00308)
Supplement: Supplementary file 1 [file DataSheet1.pdf]

Supplementary Materials for

Altered cerebral blood flow covariance network in schizophrenia

Authors: Feng Liu[1], Chuanjun Zhuo[1, 2, 3], Chunshui Yu[1]

1. *Department of Radiology and Tianjin Key Laboratory of Functional Imaging, Tianjin Medical University General Hospital, Tianjin 300052, PR China.*
2. *Department of Psychiatry Functional Neuroimaging Laboratory, Tianjin Mental Health Center, Tianjin Anding Hospital, Tianjin 300070, PR China.*
3. *Department of Psychiatry, Tianjin Anning Hospital, Tianjin 300300, PR China.*

Table S1. Brain regions with altered nodal properties of CBF binary covariance network in schizophrenia.

	P values		
Regions	Degree	Efficiency	Betweennes
**Schizophrenia > HC**			
Left inferior frontal gyrus, orbital part	-	-	0.010
Right insula	-	-	0.001
Left superior occipital gyrus	0.002	0.003	-
Left inferior occipital gyrus	0.005	0.004	0.008
Left superior parietal lobule	0.002	0.002	-
Left inferior parietal lobule	0.004	0.005	0.011
Left angular gyrus	0.005	0.007	0.011
Left temporal pole, superior part	-	-	0.003
**Schizophrenia < HC**			
Left insula	-	0.011	-
Right supramarginal gyrus	-	0.007	-
Right middle temporal gyrus	<0.001	<0.001	-
Right inferior temporal gyrus	0.002	0.003	-

Regions were considered abnormal in the patients with schizophrenia if they exhibited significant between-group differences in at least one of the three nodal topological characteristics. Abbreviation: HC, healthy controls.

Supplementary Figure S1

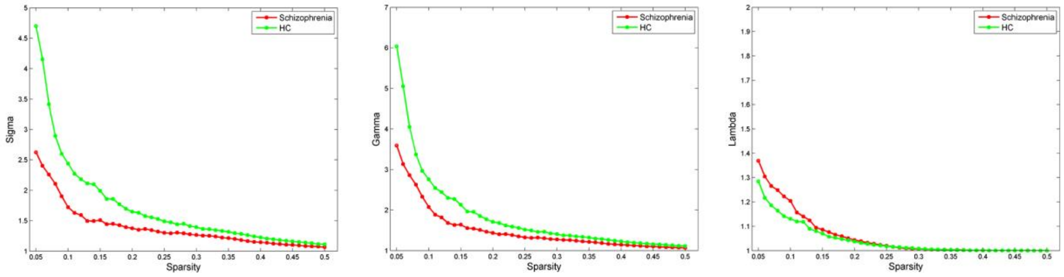

Figure S1. Small-world parameters of CBF binary covariance network as function of sparsity thresholds. Both the schizophrenia and control groups show a small-worldness (σ) greater than 1, normalized clustering coefficient (γ) greater than 1 and normalized characteristic path length (λ) approximately equal to 1, indicating that both groups exhibited a small-world topology. Abbreviation: HC, healthy controls.

Supplementary Figure S2

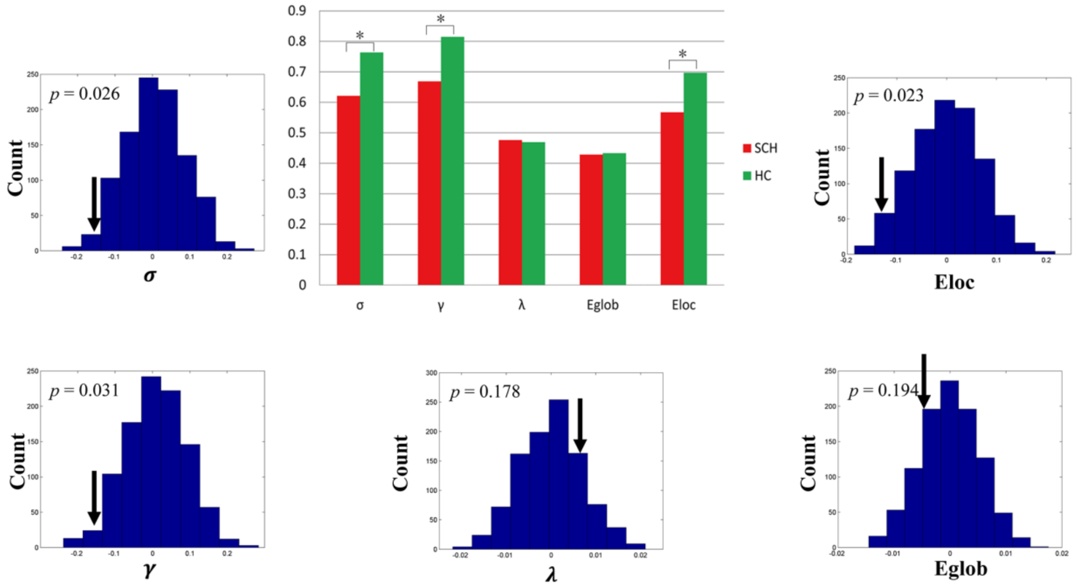

Figure S2. Global topological differences in CBF binary covariance network between schizophrenia patients and healthy controls. Each measure is expressed as the integrated area under the curve (AUC). The black stars denote statistically significant differences between the two groups (permutation test, $p < 0.05$). The histogram plot around the bar plots are null distributions of permutation tests of global network measures and the real measures are marked with black arrows. Abbreviation: SCH, schizophrenia; HC, healthy controls.

Supplementary Figure S3

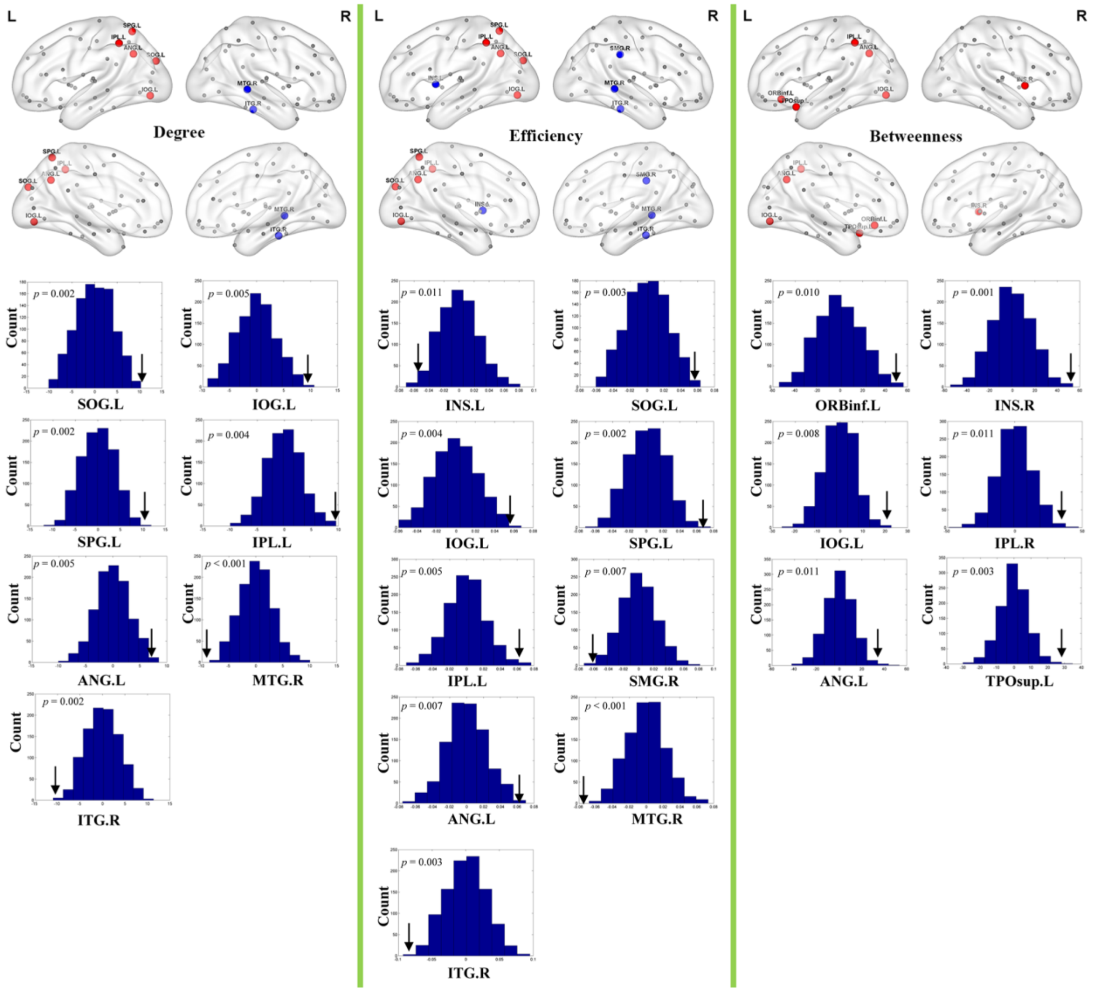

Figure S3. Nodal topological differences in CBF binary covariance networks between schizophrenia patients and healthy controls (permutation test, $p < 1/90$). Results are rendered using the BrainNet viewer (Xia, et al., 2013). Red and blue spheres represent regions with significantly increased and decreased nodal properties in schizophrenia, respectively. Grey spheres represent regions without significant intergroup difference. The histogram plots below the render plots are the null distributions of permutation tests of significant nodal network measures and the real measures are marked with black arrows. Abbreviation: ANG, angular gyrus; ORBinf, orbital part of inferior frontal gyrus; INS, insula; IOG, inferior occipital gyrus; IPL, inferior parietal lobule;

ITG, inferior temporal gyrus; L, left; MTG, middle temporal gyrus; R, right; SMG, supramarginal gyrus; SOG, superior occipital gyrus; SPL, superior parietal lobule; TPOsup, superior part of temporal pole.

Supplementary Figure S4

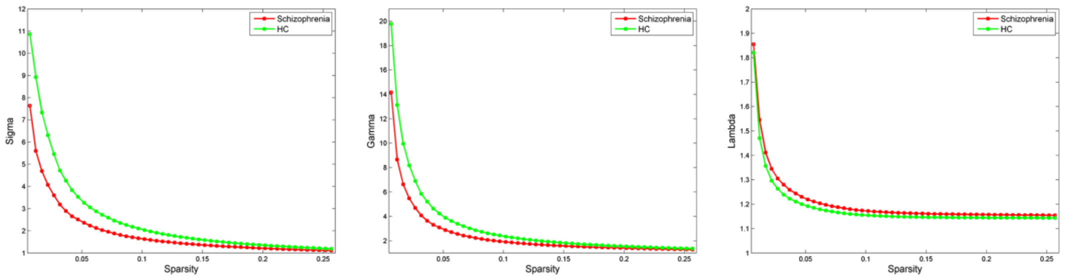

Figure S4. Small-world parameters of the high-resolution AAL-1024 CBF weighted covariance network as function of sparsity thresholds. Both the schizophrenia and control groups show a small-worldness (σ) greater than 1, normalized clustering coefficient (γ) greater than 1 and normalized characteristic path length (λ) approximately equal to 1, indicating that both groups exhibited a small-world topology. Abbreviation: HC, healthy controls. Of note, based on the criteria we used in this study, the sparsity level of AAL-1024 ranged from 0.0068 to 0.2568, with step of 0.005. Abbreviation: HC, healthy controls.

Supplementary Figure S5

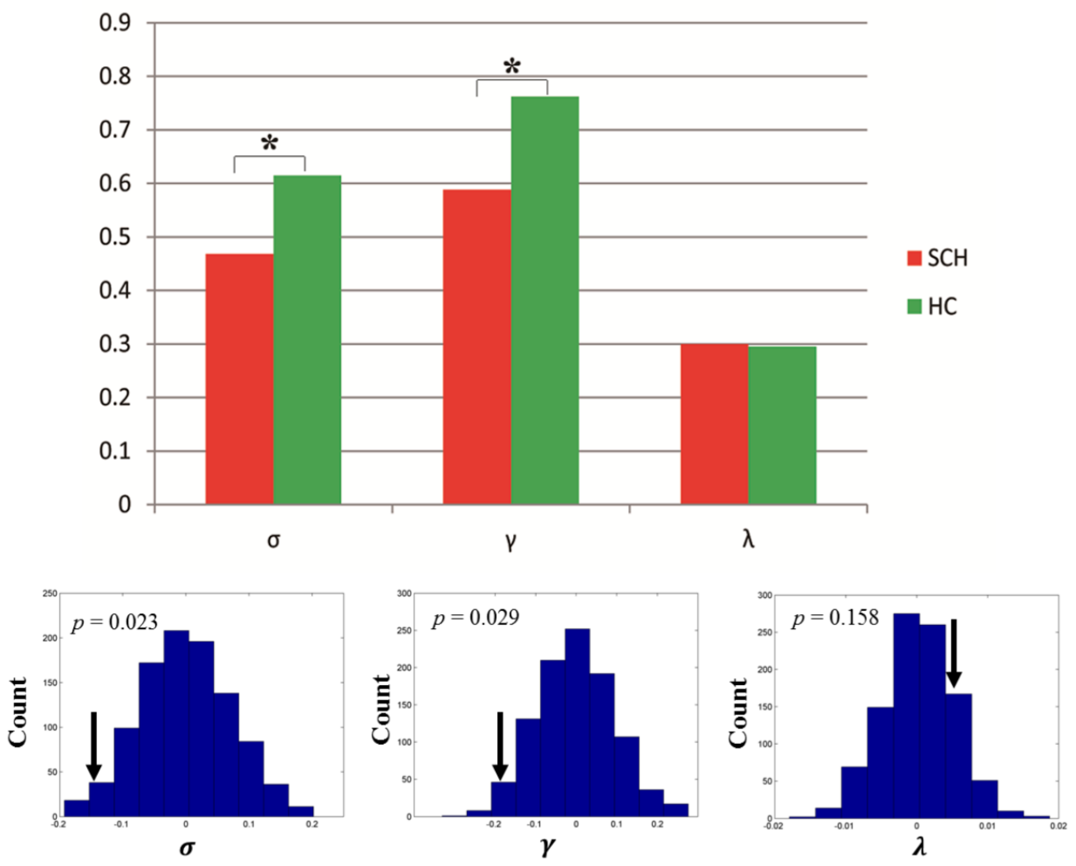

Figure S5. Global topological differences in high-resolution AAL-1024 CBF weighted covariance network between schizophrenia patients and healthy controls. Each measure is expressed as the integrated area under the curve (AUC). The black stars denote statistically significant differences between the two groups (permutation test, $p < 0.05$). The histogram plots below the bar plots are null distributions of permutation tests of global network measures and the real measures are marked with black arrows. Abbreviation: SCH, schizophrenia; HC, healthy controls.
